# Supplementary material for: The Relationship between Perceptions and Objective Measures of Greenness
Source: Int J Environ Res Public Health. 2022 Dec 6;19(23):16317. doi: 10.3390/ijerph192316317 (PMC9736070; doi:10.3390/ijerph192316317)
Supplement: Supplementary file 1 [file ijerph-19-16317-s001.zip › ijerph-2024923-supplementary.pdf]

Supplemental Table S1. Demographic characteristics of the perceived greening participants stratified by Low/Medium/High tree canopy values (n=175) within 300-m-radius circular zone surrounding residence.

Characteristic	Overall	Low Canopy ≤ 25.0 Tree Canopy	Medium Canopy 25.1 to 29.5 Tree Canopy	High Canopy > 29.5	*p* Value
Study Population	175	59	58	58	
Male	71 (40.6)	29 (49.2)	19 (32.8)	23 (39.7)	0.193
Race					0.591
White	142 (81.1)	46 (78.0)	48 (82.8)	48 (82.8)	
Black	24 (13.7)	9 (15.3)	9 (15.5)	6 (10.3)	
Other	9 (5.1)	4 (6.8)	1 (1.7)	4 (6.9)	
Hispanic	7 (4.0)	2 (3.5)	2 (3.5)	3 (5.2)	1.000
Income					0.003
less than $20,000	30 (17.1)	15 (25.4)	7 (12.1)	8 (13.8)	
$20,000-$64,999	89 (50.9)	33 (55.9)	36 (62.1)	20 (34.5)	
$65,000-$124,999	49 (28.0)	9 (15.3)	10 (17.2)	30 (51.7)	
>$125,000	3 (1.7)	1 (1.7)	2 (3.5)	0 (0.0)	
Missing	4 (2.3)				
Education					0.034
≤ High School Graduate	46 (26.3)	19 (32.2)	15 (25.9)	12 (20.7)	
2–4-year degree	98 (56.0)	32 (54.2)	37 (63.8)	29 (50.0)	
Master’s or Doctorate	30 (17.7)	8 (13.6)	5 (8.6)	17 (29.3)	
Missing	1 (0.6)	0 (0.0)	1 (1.7)	0 (0.0)	
Mother’s Education					0.376
High School GED or less	112 (64.0)	39 (66.1)	40 (69.0)	33 (56.9)	
2–4-year degree	44 (25.1)	12 (20.3)	15 (25.9)	17 (29.3)	
Master’s or Doctorate	10 (5.7)	2 (3.4)	2 (3.5)	6 (10.3)	
Missing	9 (5.1)	6 (10.2)	1 (1.7)	2 (3.5)	
Diabetes	45 (25.7)	24 (40.7)	11 (19.0)	10 (17.2)	0.005
Ever Smoked at least 100 Cigarettes	91 (52.0)	38 (64.4)	26 (44.8)	27 (46.6)	0.192
Age (Years)	50.8 (12.2)	51.5 (11.6)	50.5 (12.5)	50.5 (12.8)	0.876
Body Mass Index (kg/m2)	29.5 (6.0)	30.4 (6.0)	29.4 (6.1)	28.7 (5.9)	0.299
Systolic Blood Pressure (mmHg)	118.2 (16.9)	120.9 (14.7)	118.4 (19.5)	115.3 (15.9)	0.207
Diastolic Blood Pressure (mmHg)	79.3 (12.1)	81.1 (10.8)	80.2 (14.3)	76.8 (10.6)	0.136
Weight (Pounds)	187.3 (40.4)	193.4 (40.0)	187.5 (42.5)	181.0 (38.2)	0.252
Waist Circumference (Inches)	40.1 (6.1)	41.3 (5.4)	40.0 (6.7)	39.0 (6.0)	0.122
Hip Circumference (Inches)	43.5 (5.4)	43.9 (5.1)	43.7 (5.7)	43.3 (5.3)	0.636
Percentage Body Fat (%)	34.1 (10.6)	34.7 (10.7)	34.6 (10.5)	33.1 (10.8)	0.677

Frequencies and percentages were reported for categorical variables; means and standard deviations were reported for continuous variables. Differences in participant characteristics by tertiles of greenness metrics were tested using

Chi-square test for categorical variables and ANOVA for continuous variables. Significance was set at $p \leq 0.05$.
